# Isolation and characterization of 21 polymorphic microsatellite loci for the rockpool shrimp *Palaemon elegans* using Illumina MiSeq sequencing

**DOI:** 10.1038/s41598-018-35408-1

**Published:** 2018-11-21

**Authors:** Inés González-Castellano, Alejandra Perina, Ana M. González-Tizón, Zeltia Torrecilla, Andrés Martínez-Lage

**Affiliations:** 1 Department of Biology, Centro de Investigaciones Científicas Avanzadas (CICA) and Evolutionary Biology Group (GIBE), Universidade da Coruña, Campus da Zapateira, A Coruña, E-15008 Spain; 2 AllGenetics & Biology SL, Edificio CICA, A Coruña, E-15071 Spain

## Abstract

The rockpool shrimp *Palaemon elegans* is considered an important crustacean species within the European coastline fauna. This species is experiencing an ongoing geographical expansion beyond its native distribution range due to unintentional human introductions. A better knowledge of the genetic diversity, geographic structure and connectivity of its populations is necessary. In the present study, microsatellite loci were isolated using the Illumina MiSeq platform. The microsatellite-enriched library sequencing produced 3.9 million raw reads. Reads were processed and primer pairs were designed for microsatellite sequences amplification. Ninety-six microsatellite loci were preliminary screened in individuals from Atlantic and Mediterranean localities. From them, 21 loci exhibited reliable polymorphism and were thoroughly characterized in 30 individuals from a Cantabrian locality (Spain). No linkage disequilibrium between pairs of loci was detected. Number of alleles per locus ranged from 2 to 12. Observed and expected heterozygosities ranged from 0.033 to 0.833 and from 0.033 to 0.869 respectively. No significant departure from the Hardy-Weinberg equilibrium was detected in most of loci. This is the first time that microsatellite markers have been developed for *P*. *elegans*. This characterized microsatellite suite provides new suitable tools for further analyses, facilitating the understanding of population genetics both in natural and introduced populations.

## Introduction

The rockpool shrimp *Palaemon elegans* Rathke, 1837 is a crustacean decapod with a native geographical distribution ranging from the Atlantic Ocean (from Scotland and Norway to Mauritania, including the Azores, Madeira and Canary Islands) to the entire Mediterranean Sea and the Black Sea^1^. Nowadays, this shrimp also inhabits the Caspian Sea and the Aral Sea because of unintentional introductions^2^. Similarly, this species was introduced in the Baltic Sea where it is replacing the native congeneric *P*. *adspersus*^2,3^. Occurrence of *P*. *elegans* in the northeast coast of the United States was recently reported^4^, so its distribution range is currently extended beyond European waters.

This shrimp is common in tidal rockpools and in *Zostera*, *Posidonia* and *Cymodocea* sea grasses meadows. It also can be found in hypersaline lagoons and in slightly brackish water close to river mouths^5^. *Palaemon elegans* is characterized by its capability to adapt to highly variable environmental conditions. Therefore, due to its broad ecological niche and its recent and ongoing geographic expansion, it is considered an important species within the European coastline fauna^6^.

Population genetics analyses in *P*. *elegans* are scarce and exclusively based on mitochondrial DNA (mtDNA) markers. Reuschel *et al*.^6^ carried out a phylogeographic analysis with two mtDNA markers, revealing the existence of three haplogroups, one of them in the Atlantic localities (type I) and two from the Mediterranean localities (types II and III). Genetic differentiation between the Atlantic populations (type I) and the Mediterranean populations (type II) was observed as well as the putative occurrence of a cryptic species within *P*. *elegans* (type III). This population genetic pattern was supported again with mtDNA markers in later phylogeographic studies in the Mediterranean Sea^7,8^. These findings highlighted the need to carry out further genetic studies with polymorphic nuclear markers, such as microsatellites, in order to clarify the population biology of *P*. *elegans*.

Given its codominant nature, biparental mode of inheritance and high levels of polymorphism^9^, microsatellite markers have been used in a wide range of applications in population genetics, ecological, conservation and evolutionary studies^10^. Indeed, microsatellites loci are extremely valuable tools in population genetics because they might reveal the existence of genetically isolated populations even in fine-scale studies^11^. Polymorphic microsatellite loci have been developed for three *Palaemon* species^12–14^. In the case of *P*. *serratus*^14^, thirteen loci showed positive cross-species amplification in *P*. *elegans*. Nevertheless cross-amplification from congeneric species is not generally feasible because inherent problems like allele size homoplasy, polymorphisms biases, null alleles presence, broken repeats motifs or amplification of non-orthologous loci could arise^15–18^. Thus, de novo development of species-specific microsatellite markers is strongly recommended.

Classically, microsatellite development required the construction of an enriched library by cloning and Sanger sequencing, a laborious, time-consuming and expensive strategy^19,20^. This drawback could be overcome with the advent of next-generation sequencing (NGS) technologies, which produce a large amount of sequences, providing a faster and cost-effective approach for microsatellite loci discovery^21^. The first microsatellite markers developed using NGS were based on Roche 454^22^, however Illumina has demonstrated its capability for microsatellite isolation^23–25^ and it is currently the platform used for this purpose.

The aim of this study was the isolation and characterization of novel polymorphic microsatellite loci for *P*. *elegans* using Illumina high-throughput sequencing. Given that there are no microsatellite loci previously developed for this shrimp species, these markers will provide new suitable tools in order to assess the genetic diversity, geographic structure and connectivity of populations at smaller geographic scales, among other possible future applications.

## Results and Discussion

The development of microsatellite markers in non-model species has become a rapid and cost-effective process thanks to the advances in high-throughput sequencing technologies^26^. Thousands of microsatellite loci can be identified in the large amount of sequence data. Illumina is currently the platform used to accomplish microsatellite isolation and it produces more reads at lower prices than Roche 454^27^. Paired-end sequencing is frequently the preferred strategy for microsatellite isolation as longer reads are generated. In this study the *P*. *elegans* microsatellite-enriched library was sequenced using Illumina MiSeq PE, an approach already used for microsatellite discovery in other organisms, e.g. Ewers-Saucedo *et al*.^28^, Landínez-García & Márquez^29^ and Gaeta *et al*.^30^. Here, the microsatellite-enriched library sequencing produced 3,902,540 paired-end reads. These paired-end reads were processed and overlapped into 1,766,031 sequences, with a mean length of 171 bp (range: 50–538 bp). Tandem repeats were identified and 500 sequences containing microsatellite motifs were used for primer design. For the preliminary screening, ninety-six out of these 500 primer pairs were tested in five individuals from the different Atlantic and Mediterranean localities that already showed genetic divergence with mitochondrial markers in Reuschel *et al*.^6^. From them, primer pairs that produced no amplification or unexpected size PCR products were discarded. Likewise, monomorphic loci were excluded, as well as loci that showed stuttering patterns of ambiguous interpretation. A final suite of 21 microsatellite loci (perfect tri- and tetranucleotide repeats) yielded consistent amplification and reliable polymorphism. Sequences containing these 21 microsatellite markers were deposited in GenBank under accession numbers MH078079-MH078099 (Supplementary Material S1).

The microsatellite loci were characterized through the genotyping of 30 individuals collected in the Santoña locality (Table 1). Successful amplification and scoring was achieved for the 21 microsatellite loci in all individuals. No evidence of linkage disequilibrium between pairs of loci was detected after Bonferroni correction. Hence, all microsatellites were considered as independent markers. Regarding to the genetic variability, the number of alleles per locus ranged from 2 to 12, with a grand mean of 4.6 alleles per locus. The observed heterozygosity (Ho) for each locus ranged from 0.033 to 0.833, and the expected heterozygosity (He) for each locus ranged from 0.033 to 0.869 (Table 1). Ho and He averaged 0.390 and 0.464, respectively. These levels of polymorphisms are in line with those found in the counterpart common littoral shrimp *P*. *serratus* in other Atlantic locality^14^. Significant departures from Hardy-Weinberg equilibrium (HWE) were found in four loci (Pe11, Pe14, Pe18 and Pe19) after sequential Bonferroni correction (Table 1). A heterozygote deficit was detected for these four loci. Similarly, heterozygosity deficiency has been reported in other shrimp species^12,14,31–33^. Among the frequent causes for HWE deviations, inbreeding is quite common. The four loci out of HWE were accompanied with positive FIS values (Table 1). A global FIS value of 0.174 also suggested a heterozygosity deficit in the Santoña locality. However, null alleles could also be invoked to explain heterozygote deficits. Although microsatellite null alleles are widespread, marine invertebrates have demonstrated particularly higher frequencies than other groups^34,35^. Due to the instability of the flanking sequences of microsatellites, some alleles could not be amplified and consequently dropped out, resulting in a homozygote excess. The four loci that deviated from HWE showed evidence of occurrence of null alleles even though mainly in a low proportion (Table 1). Thus, presence of null alleles is the most likely explanation for those departures from HWE. Overall, most of the 21 loci showed low null allele frequencies (Table 1). Only one locus, the locus Pe11, exhibited a high null allele frequency, >0.2 according to Chapuis & Estoup^36^. This locus Pe11 is precisely one of the four loci that showed significant deviation from HWE. Therefore, given that the locus Pe11 could be potentially problematic, its inclusion in future analyses might be carefully considered.

**Table 1:** Characterization of the 21 microsatellite loci for *Palaemon elegans*. 5′ tails attached to reverse primers are in brackets.

Locus name	Accession no.	Primer sequence 5′-3′	Dye	Repeat motif	N	Na	Allele size (bp)	Ho	He	*P*HWE	FIS	Null
Pe01	MH078079	F: GCTCAAGGACACCGCTAACT	HEX	(AAC)8	30	3	125–137	0.267	0.376	0.099	0.306	0.096
		R: [M13]CGCTCGCATGTCTATGGTCT										
Pe02	MH078080	F: GGAATGGTGGCCTGAATGGA	6-FAM	(ATT)8	30	4	138–147	0.667	0.639	0.958	−0.026	0.000
		R: [CAG]CTGTTGGAGCCCTTGGACTT										
Pe03	MH078081	F: TGATGACTGGTGCGGTGTAC	6-FAM	(GAT)9	30	3	111–120	0.500	0.473	0.975	−0.041	0.000
		R: [CAG]CCGGGCTATCGTCATCATCC										
Pe04	MH078082	F: GCCTCAGGTCTTCATCAGGG	6-FAM	(GGA)8	30	2	102–105	0.567	0.499	0.461	−0.118	0.000
		R: [CAG]TCTTTGGGCTGGTCATCGTC										
Pe05	MH078083	F: TCAACCCTAGGTCTGAGCCA	6-FAM	(CTT)4	30	2	195–210	0.233	0.255	0.642	0.102	0.027
		R: [CAG]TCTTGGCCGCTGATGCTATT										
Pe06	MH078084	F: AGCTACTGGACCGCTCGATA	6-FAM	(AGT)7	30	9	157–196	0.600	0.708	0.994	0.169	0.060
		R: [CAG]CTGTTGCAATTGGGATCGGC										
Pe07	MH078085	F: CATTGTACCATTGACCGGCG	6-FAM	(CGCT)4	30	4	100–132	0.467	0.658	0.028	0.307	0.105
		R: [CAG]GCTCATCCTGATCCTGACCG										
Pe08	MH078086	F: TTCCATTCGAGGATCTGCGG	6-FAM	(GCTT)5	30	3	314–370	0.167	0.212	0.295	0.229	0.064
		R: [CAG]TCTGCGAACTTCGGTTACGG										
Pe09	MH078087	F: TGTCTGAGGCCTTTGTCCTG	6-FAM	(GAT)7	30	2	228–237	0.033	0.033	0.926	0.000	0.000
		R: [CAG]GGCGACAAGAATGCTTCGTC										
Pe10	MH078088	F: GGAGGGAGTGCAATGAGGTT	6-FAM	(AAG)14	30	9	149–179	0.767	0.816	0.002	0.078	0.018
		R: [CAG]GTGGGATCAGGACTCGAAGC										
Pe11	MH078089	F: GGTGTTTAGCTGGTGGACCA	6-FAM	(TGA)8	30	10	123–156	0.300	0.742	0.000*	0.606	0.264
		R: [CAG]CCGGCATCTCCACCATTAGT										
Pe12	MH078090	F: TTGCAGTGGCGTGACAAGAT	HEX	(TGA)8	30	4	116–143	0.233	0.385	0.005	0.408	0.122
		R: [M13]GGTCGGATGTAACTGCAGCT										
Pe13	MH078091	F: AAGTTGCTCCAACCGAGTCA	6-FAM	(TTG)6	30	3	195–204	0.167	0.209	0.554	0.220	0.059
		R: [CAG]CGTCCCTAATGCAGCTTCCT										
Pe14	MH078092	F: GTCACGTTGCACGAATGTCC	6-FAM	(ACT)19	30	12	131–179	0.833	0.869	0.000*	0.058	0.040
		R: [CAG]CCGTACTGGCCACAACGAAT										
Pe15	MH078093	F: CCCTTCCCTTTCCTCAACGA	HEX	(TCA)7	30	3	140–146	0.433	0.539	0.588	0.213	0.058
		R: [M13]GTCTTCGTCCGTCCTCCATC										
Pe16	MH078094	F: TACTTGGAAGGTCAGGCAGC	HEX	(AAG)13	30	4	163–175	0.267	0.388	0.122	0.329	0.116
		R: [M13]CCCATTCTCCCTTCCACTCC										
Pe17	MH078095	F: CCTACGATGTCAGGATGCCA	HEX	(AATC)11	30	8	122–158	0.767	0.712	0.048	−0.060	0.011
		R: [M13]CACTCTCGCTCATCTTGGCT										
Pe18	MH078096	F: TATTTACCTGCGAGTGCGGT	HEX	(AAG)6	30	3	213–222	0.233	0.263	0.000*	0.129	0.069
		R: [M13]AGCCAGTTGACGACGTTGTT										
Pe19	MH078097	F: GAGAAGACTCGGTGTGGCTG	6-FAM	(AGG)9	30	4	101–113	0.333	0.406	0.000*	0.196	0.062
		R: [CAG]GCGAATCACACTTGGCCTCT										
Pe20	MH078098	F: GCACAGAGCCTTATCTCCCTC	HEX	(ATC)6	30	2	262–265	0.067	0.180	0.001	0.640	0.141
		R: [M13]AGGACATCTTGGTGGCCATG										
Pe21	MH078099	F: GCAAAGGCAGACCATCATGC	6-FAM	(TGTC)5	30	2	219–223	0.300	0.375	0.273	0.216	0.064
		R: [CAG]GCGTTAGTCCTTCTGCGAAG										

Notes. N, sample size; Na, number of alleles; Ho, observed heterozygosity; He, expected heterozygosity; *P*HWE, Hardy-Weinberg equilibrium *p*-values; FIS, inbreeding coefficient; Null, null allele frequency.

*Significant departure from HWE after the sequential Bonferroni correction (*P* < 0.00048).

Microsatellite markers have proven to be crucial in understanding population genetics and ecology of several shrimp species as *Penaeus setiferus*^37^, *P*. *vannamei*^38^, *P*. *monodon*^39^, or *Aristeus antennatus*^40^. Assessment of genetic diversity and population structure provides interesting data for conservation and fishery management measures. The microsatellite loci characterized here provide new suitable tools available for the scientific community to study *Palaemon elegans* population dynamics. Specifically, future studies using these microsatellite loci will shed light about *P*. *elegans* phylogeography at smaller geographic scales. In fact, there are ongoing projects that aim to analyze Atlantic and Mediterranean localities along the European coastlines using these markers to address the genetic differentiation between these basins. This microsatellite set will be also greatly useful for inferring the introduction routes and the genetic profiles of the introduced populations of this species in European and North America waters^41–43^.

## Material and Methods

Specimens of *P*. *elegans* used in this study were collected from Ártabro Gulf (Galicia, Spain, 43°18′48.1″N, 8°33′51.3″W) in 2012 and ethanol-stored at laboratory. Morphological identification was carried out according to González-Ortegón & Cuesta^5^. Genomic DNA was extracted from 25 mg of muscle abdominal tissue using NZY Tissue gDNA Isolation kit (NZYTech) following the manufacturer’s instructions. Extracted DNA quality and concentration was determined with the NanoDrop ND-1000 spectrophotometrer (Thermo Fisher Scientific).

A microsatellite-enriched genomic library from this sample was constructed at AllGenetics & Biology SL (A Coruña, Spain). The library was prepared using Nextera XT DNA Library Preparation kit (Illumina) following the manufacturer’s instructions. Microsatellite motifs AC, AG, ACG and ATCT were used for the library enrichment. The enriched library was sequenced in the Illumina MiSeq PE300 platform (Macrogen Inc., Seoul, Korea). Reads were processed using Geneious 10.0.5^44^ and in-house developed scripts. Sequences containing microsatellite loci were selected for primer design. Primer pairs were designed using Primer3^45^ for PCR amplification of 500 microsatellite loci, with primers hybridizing at the flanking regions of tandem repeats.

A preliminary panel of 94 primer pairs was screened in individuals from five different Atlantic and Mediterranean localities (one individual per locality): Ré Island (France, 46°11′36.2″N, 1°30′10.2″W), Santoña (Spain, 43°28′04.2″N, 3°28′30.6′′W), Benijo (Spain, 28°34′21.7″N, 16°11′47.0″W), Llança (Spain, 42°21′06.3″N, 3°11′15.1″E) and Collioure (France, 42°31′43.8′′N, 3°05′07.6′′E). All genomic DNA extractions were accomplished following the aforementioned protocol. PCR reactions were performed following Schuelke^46^ so for each microsatellite locus three primers were used: a specific forward primer, a specific 5′-tailed reverse primer and a fluorescently-labeled oligonucleotide identical to the 5′-tail of the reverse primer. As 5′-tails we used the universal sequences M13 (5′-GGAAACAGCTATGACCAT-3′) and CAG (5′-CAGTCGGGCGTCATCA-3′). M13 oligonucleotides were labeled with the HEX dye, meanwhile CAG oligonucleotides were labeled with the 6-FAM dye. Nested PCR reactions were conducted in a final reaction volume of 12.5 µL, containing 1 µL of DNA (10 ng/µL), 6.25 µL of the Type-it Microsatellite PCR Kit (Qiagen), 4 µL of PCR-grade water, and 1.25 µL of the primer mix (2 µM for both forward primer and HEX-M13 or 6-FAM-CAG oligonucleotides and 0.2 µM in the case of M13- or CAG-tailed reverse primers). The optimal PCR conditions consisted in an initial denaturation step at 95 °C for 5 min, followed by 30 cycles of 95 °C for 30 s, 56 °C for 90 s, 72 °C for 30 s; 8 cycles of 95 °C for 30 s, 52 °C for 90 s, 72 °C for 30 s; and a final extension step at 68 °C for 30 min. Amplification reactions were performed on a T100 Thermal Cycler (Bio-Rad). Fluorescently labeled PCR products were run on a 3130xl Genetic Analysis System (Applied Biosystems) for fragment analysis in the Scientific Research Support Services (University of A Coruña), using GeneScan 500 (−250) ROX internal size standard (Applied Biosystems). Geneious 8.0.5^44^ was used for fluorescent profiles analyzing and allele peaks calling.

All microsatellite loci that yielded consistent amplification and reliable polymorphism were further assessed by genotyping 30 individuals from the Santoña locality (Cantabria, Spain). Singleplex PCRs were carried out as mentioned above. For each locus characterization, number of alleles, observed heterozigosity (Ho), expected heterozigosity (He) and deviations from Hardy-Weinberg equilibrium (HWE) were estimated using GenAlEx 6.5^47^. GENEPOP^48^ was used to estimate inbreeding coefficient (FIS) and to test linkage disequilibrium between pairs of loci. Significance level was adjusted applying the sequential Bonferroni correction for multiple testing. Null allele frequency was estimated using FreeNA^36^ and the EM algorithm^49^.

### Accession codes

Sequences containing the microsatellite loci were deposited in GenBank under accession numbers MH078079-MH078099.

## Electronic supplementary material

Sequences contained the 21 microsatellites of Palaemon elegans

## Data Availability

The datasets generated and/or analyzed during the current study are available in the GenBank repository (accession numbers MH078079-MH078099) but restrictions apply to the availability of these data, and so they are not publicly available until article publication. Data are however included as Supplementary Information files (Supplementary Material S1).
