# Supplementary material for: Isolation and characterization of 21 polymorphic microsatellite loci for the rockpool shrimp Palaemon elegans using Illumina MiSeq sequencing
Source: Sci Rep. 2018 Nov 21;8:17197. doi: 10.1038/s41598-018-35408-1 (PMC6249324; doi:10.1038/s41598-018-35408-1)
Supplement: Supplementary file 1 — Sequences contained the 21 microsatellites of Palaemon elegans [file 41598_2018_35408_MOESM1_ESM.pdf]

Isolation and characterization of 21 polymorphic microsatellite loci for the rockpool shrimp *Palaemon elegans* using Illumina MiSeq sequencing

Inés González-Castellano[1], Alejandra Perina[1,2], Ana M. González Tizón[1], Zeltia Torrecilla[1], Andrés Martínez-Lage[1*]

Supplementary Material S1. Sequences contained the 21 microsatellites of *Palaemon elegans*.

>*Palaemon elegans* microsatellite DNA locus Pe01

GTTTCAGACATTTAGTAACACATAACGGGTCCGCTTGAGCAGCAAGACACCGCTCAAGGACACC
GCTAACTTACAGACAC**AACAACAACAACAACAACAACAAC**AGATAAAACCGCTGTTGACTAGAT
GGTGAAAGACCATAGACATGCGAGCGTGGAAATCGTGATGATCGGTTGAATTAAAGAATTAGAA
AGTGGAGCTTTGTGATCGCTTATGCAAAACCCATTTG

>*Palaemon elegans* microsatellite DNA locus Pe02

GTGCAAGATAACGGGGAATGGTGGCCTGAATGGAAGTTTAGTGTTCAGGGATTGTTC**ATTATTA**
TTATTATTATTATTATTGCTAGCTAGGCTACAACCCCAATTGGAAAAGCAGGATGCTATAAGTC
CAAGGGCTCCAACAGGGAAAAAC

>*Palaemon elegans* microsatellite DNA locus Pe03

ACTTCTACCTCTTTCAAATTTTATCAACTTTCTCCAACATTGCTGTCTGACCTCTCACCCCCAT
TTTCTCGGCGTCAATGACTGTATCAGGACTTGGATTCGGTTATTTCTAAAAACATAATCTGGCA
GTTGATGACTGGTGCGGTGTACGGAATTGTTTAGAATGTTCTTTT**GATGATGATGATGATGATG**
ATGATGATGACGATGATAATAGTGAGGATGATGACGATAGCCCGGAGACAGGAAGTGATTGTGC
TCTCTGAAAT

>*Palaemon elegans* microsatellite DNA locus Pe04

GGGCCTCAGGTCTTCATCAGGGTTGGATCTCTGGCCGTTGTC**GGAGGAGGAGGAGGAGGAGGAG**
GAAAACACTGACGATGACCAGCCCAAAGAAGACCAACCAC

>*Palaemon elegans* microsatellite DNA locus Pe05

CTCTCTCTCTCTCTCTCTCTCTCTCTCTCTCTCTCTCTCTCTCATACTGTAATGTACTGT
ATATCTATCAACCCTAGGTCTGAGCCATCTTAGTTTGGTATGTTCTAGACGGGTAAGGATGTCC
TTTACGCCTAAAAGAATTTCCCTTTTTT**CTTCTTCTTCTT**CGGCAGCGTGCAATCTCGTGCCAG
GAGGAGAAGGCTCACAATTACCGTTTTCTAACTCTGGGAAGCAACGGCGATATAAATAGCATCA
GCGGCCAAGATAAGAGTTCTAGCTATTGCGATGACCAGAAACGAGCGTTGATGATGGTGATAGC
GCCGAGGAAAGTAG

>*Palaemon elegans* microsatellite DNA locus Pe06

AGCTACTGGACCGCTCGATACGAGGGTTACACAGCTCCACGCTGTACCTGTG**AGTAGTAGTAGT**
AGTAGTAGTAGCAGCAGTAGCAGTGGAAGTAGTAATGGTAATAGAAAGAACTGTAATGCCGATC

CCAATTGCAACAGCAGCATTAACATTAGCATCAACAAGAAGAATAACAATAAAAATAACAGTAA
TAATAACATTACCTAATTCTAGCCCCATTTTCAGACTGCCTCCATGAAAT

>*Palaemon elegans* microsatellite DNA locus Pe07

CCAACATTGTACCATTGACCGGCGCTTACTACTTTCACCGAAGCTGGG**CGCTCGCTCGCTCGCT**
CCGACAATAGTGCCCTGACACTGAGACACCAAACGGTCAGGATCAGGATGAGCCTTTGAG

>*Palaemon elegans* microsatellite DNA locus Pe08

GCAGTTCCTGTTCCATTCGAGGATCTGCGGAGTAGTTCACAGGGTAGTGCTCCTTCAACACGCT
CTCCCCTTGGGATTAGTCTGTACGCTGAGCTTGGGATAGATACCTCTATCTTCACCCTTCTAAG
CTCACTGACCCTTCGGGGATCTTCGGCAGACACTCTCGCAAGTACGAAAGATTGTCTTCACCTG
GGAGTTCTTCTCCCCAGTTAGCTCAGAAGCGCCAGAGCGACTTGTTACCATTGTGCTCTTGGTC
TTCGGAGCTGAGCTCTCCGAAGTTC**GCTTGCTTGCTTGCTTGCTT**GAAGATTGCCCGGCCTTGT
GCCAAACACGGGCTCTTGCTTTCGCAGCCCGTAACCGAAGTTCGCAGA

>*Palaemon elegans* microsatellite DNA locus Pe09

AGAGTGGAGGGGACATGTCTGAGGCCTTTGTCCTGCAGTGGACTAGCAACG**GATGATGATGATG**
ATGATGATGAGGCTTAGCAAAGTAGGCTTATCATGTGACTTACATTACTTCTGCATTCTATTAG
TGCCACCAGTTTTGCTGTACGGTAGGTATTCTTGTTATAGTAATGGTAACCATTTCACAAGGAG
TTTACATTTTCTGAAAATAATGTCTGACGAAGCATTCTTGTCGCCATATTCAAAATGAAATTAT
A

>*Palaemon elegans* microsatellite DNA locus Pe10

AACAACGGGGGGTGAGGGGGGGAAGGAACCCCCACAGGGATATTACGGGGGGTCCTAGGGTGAG
ATGGGAGGGAGTGCAATGAGGTTGAAAATGAGCAAACACTGGGAAGACCAACATTGAGGA**AAGA**
AGAAGAAGAAGAAGAAGAAGAAGAAGAAGAAGAAGAAGACGACGGTGATTGCCAGAAACAAGTG
TTTGGTTTTGCTTCGAGTCCTGATCCCACC

>*Palaemon elegans* microsatellite DNA locus Pe11

CTTGATGAGGAACACGGGTGTTTAGCTGGTGGACCATATGATGGTCGGTGG**TGATGATGATGAT**
GATGATGATGAAGAGGAGAGTTGCTAATGGAGATGATCAACACTAATGGTGGAGATGCCGGAGG
TTGTCGTTGGAGTAGATATTGATGTATTAGGGGATGTAAAGGCAATATCTTATGATAGAGAGGA
TATCTGATGATAAAGTAGGTCCAATTGGTGATGCAGAAGGGGATTGGTAAAAGAATAAAAGGAT
GATAGATATTGATGGATATTGATGGATAAAAAAGGGCTGGAGAGGTAATGACGTCAGCACTAAT
AGGTCACAGCCATAAACGGATAATATGGAAATTATAAACAC

>*Palaemon elegans* microsatellite DNA locus Pe12

GCCTATTGCAGTGGCGTGACAAGATA**TGATGATGATGATGATGATGATGA**CTCTAGTGTCAGTT
TATGAGTTATACCAATTGCTATTTAAATAATAAGAGCAGCTTTTCTTTATCAGCTGCAGTTACA
TCCGACCAAAATACATAATACTTAATCTTAGGCTACACCATTATAC

>*Palaemon elegans* microsatellite DNA locus Pe13

AAGTTGCTCCAACCGAGTCATTTTACCTC**TTGTTGTTGTTGTTGTTG**AGTGATAACCGAGGATT
TTCTACCTGAGAACTTTAGCGTTCATCATATCCTTCTATGAGTTTCCAAAATGAAACAAAATGT
ACGGTCGTCAAGGTGACCGAGTATCATAAGGAAGGAAGCTGCATTAGGGACGTGACATTGCCAT
GTCGCCAAGAAGCACTTAGTCGAGAGGTAC

>*Palaemon elegans* microsatellite DNA locus Pe14

AAATACTCATTTGAATATAAAAGCTGAGATGATTTCCACATTTATATTCGCTGCAAAACTCTCA
TACAAGTCACGTTGCACGAATGTCCTAGATTGGGCCTTGGTAAGGCTTGTCTTCCT**ACTACTAC**
TACTACTACTACTACTACTACTACTACTACTACTACTACTACTACTACTACTAGAACTCTGTGTATT
GGATAGCCATTCGTTGTGGCCAGTACGGCCATTCAGCAGCAT

>*Palaemon elegans* microsatellite DNA locus Pe15

ACTTTTCCCTTCCCTTTCCTCAACGAAGAAAGCTCAAATCACAGGGAACAC**TCATCATCATCAT**
CATCATCACGATGCGTCTCGGCGAGACACCACCAACAGGAAGGATGGAGGACGGACGAAGACGA
ATCAGGTGATTTTGGAGAACTAAATTTTTGGTCACCGATTACGAAGCAGTTGTTTTCAACATGA
TGCTCCAAAAGCG

>*Palaemon elegans* microsatellite DNA locus Pe16

GTCTTAATGTTTTCATTGCTGAGTCTCTCAGACTACTTGGAAGGTCAGGCAGCTATTCACCTCT
TGTGGCAAT**AAGAAGAAGAAGAAGAAGAAGAAGAAGAAGAAGAAGAAGAAG**ATGAGGAATGGGAAAA
GGGAAACGAGACACAGGAGGTGCATAATGAAGCTGAGGAAAAGATGGAGTGGAAGGGAGAATGG
GAGAATTTAATGAAGTTGAAATAATTCCCTCAAGGGAAAAAAGGATTATATTTGCGACACCACT
AATTCAAAATAAATAAAAAAATAATAATAAAAATAACAATAAATTTATGCATACCTATCTGTCT
ATCAGTTTATGTATACGAA

>*Palaemon elegans* microsatellite DNA locus Pe17

CCTACGATGTCAGGATGCCAGAAAACTTGAAATCAGTC**AATCAATCAATCAATCAATCAATCAA**
TCAATCAATCAATCAATCAATTGTCACAGCTGAATTACTAGCCAAGATGAGCGAGAGTGAGGGT
TTCACATTCACACCGCTAGATGGCGGTGGGGTAGCAG

>*Palaemon elegans* microsatellite DNA locus Pe18

GTTCCTTCCAAGTCCGCCGTTTCGGATATTTACCTGCGAGTGCGGTCGGAACTCCCGTCGAATC
GCATGTCGATTTCGCCCGAGATGAACGAGAACTTGAAGCGCCTTCCCGTCGAAGAGCGAGATCA
GGGATGCGAAAGCAAGGAGCTCGCGACGGCG**AAGAAGAAGAAGAAGAAG**ATTCAGAAGGAGGCG
TCGGCGAAGAGCATTGACAACAACGTCGTCAACTGGCTCCCAACGCC

>*Palaemon elegans* microsatellite DNA locus Pe19

GGAGAAGACTCGGTGTGGCTGTGG**AGGAGGAGGAGGAGGAGGAGGAGGAGG**CGTTTGGTATGGG
GACTAGAGATACACAGAGGCCAAGTGTGATTCGCTTTTACC

>*Palaemon elegans* microsatellite DNA locus Pe20

ATCCAATCGCGGGCACAGAGCCTTATCTCCCTCTTCGTCTGAACTACAAATGTCACTACCGTCA
TTAGTTACTTTAGTGAGGGTTTGTAAACTCTTTCTCCTTTCCAGGTCATTATCTTT**ATCATCAT**
CATCATCATCATTATTACCTTCATCAACTTCATCCTCGGGTTCGGAAACGCTGTCGACGAAGTT
CAACAGCTCTGTGAGTGAGCTGCCTATACTTTCAAAACTTGCCTTCATGGCCACCAAGATGTCC
TCTTCTCCTGG

>*Palaemon elegans* microsatellite DNA locus Pe21

GTGTGTCTGTCAAGCCAATTTGTCATCACAGCCCCTCTTGCAAGGTTGAAAGGATAATCTGTCA
AACTTACTGCAAAGGCAGACCATCATGCATTTATGTGCGTGAAGGGAGATCGTCCAATAGGTCC

GTT**TGTCTGTCTGTCTGTCTGTC**AGTATGCTGTCCATTTACCCTTTTCCAGTCTGATGAGACTA
AGGATAAGATAAAATCGTGCAAGACCGCAGACGTTTTACCCGACGCGTCGTGTGGGGATTTCTC
AACCTTCGCAGAAGGACTAACGCTGGCGGG
